# Right aortic arch with common origin of right carotid and left innominate artery: A case report

**DOI:** 10.1002/ccr3.6532

**Published:** 2022-11-10

**Authors:** Swati Chand, Omofolarin Babayale, Devesh Rai, Sangharsha Thapa, Sangam Shah, Jeremiah P. Depta, Bipul Baibhav

**Affiliations:** ^1^ Rochester General Hospital Rochester New York USA; ^2^ University of Minnesota Medical School Minneapolis Minnesota USA; ^3^ Institute of Medicine Tribhuvan University Kathmandu Nepal

**Keywords:** aortic arch, carotid, common origin, innominate

## Abstract

Aortic arch anomalies are rare congenital malformations with an incidence of approximately 1–3%. Right aortic arch is an anatomical variant with an incidence of <0.1% associated with various congenital heart diseases. We present a case of a 26‐year‐old female patient with a right aortic arch with a common origin of right carotid and left innominate artery.

## Abstract 2

We report the first case in the literature of a right‐sided aortic arch with a common origin of right carotid and left innominate artery.

## 1 INTRODUCTION

Right aortic arch (RAA) is a rare anatomical variant with an incidence of <0.1% and is associated with various congenital heart diseases.^1^, ^2^ The patient can be asymptomatic and present at any age with abnormal chest radiography or have symptoms like dyspnea, dysphagia, and signs of congestive heart failure. RAA is commonly associated with congenital heart disease. Computed tomography (CT) and magnetic resonance imaging (MRI) are the diagnostic modalities to evaluate the anatomic anomalies of aortic arches.^3^ Based on regression of aortic arches leading to various branching patterns during development and the presence of other associated heart defects, the right aortic arch is classified into three types.^4^ (Table 1) We present a case of the right aortic arch with a common origin of the right carotid and left innominate artery, which has not been described in literature before.

**TABLE 1:** Types (Figure 2), mechanism, and its clinical correlate of right‐sided aortic arch

Types	Mechanism	Associated disease
Type 1: right‐sided aortic arch with mirror image branching: the major arteries branching out from the arch are the left innominate artery, followed by the right common carotid and right subclavian arteries	Regression of the left fourth branchial arch between left ductus and dorsal aorta.	Cyanotic congenital heart disease like Tetralogy of Fallot and truncus arteriosus
Type 2: right‐sided aortic arch with aberrant left subclavian artery	Abnormal involution of left fourth arch in between the left subclavian and left common carotid artery	Associated with Kommerell's diverticulum and is rarely associated with other congenital heart diseases
Type 3: right‐sided aortic arch with isolation of the left subclavian artery	Regression of embryological left arch at two segments on either side of left subclavian artery	Subclavian steal syndrome and vertebrobasilar insufficiency.

## 2 CASE PRESENTATION

A 26 years‐old female patient presented with a complaint of gradually worsening shortness of breath for a month. Shortness of breath was worse on exertion and relieved with rest. She occasionally had escaped beat. The patient denied complaints of orthopnea, paroxysmal nocturnal dyspnea, chest pain, cough, dizziness, syncope, leg swelling, recent travel, immobilization, fever, and chills. Past medical history was significant for tetralogy of Fallot (TOF), patent foramen ovale (PFO), left pulmonary artery stenosis, and bilateral hearing loss. She had surgical repair of TOF, PFO, and stenting for pulmonary artery stenosis at a young age. Family history was unremarkable.

**FIGURE 1:**
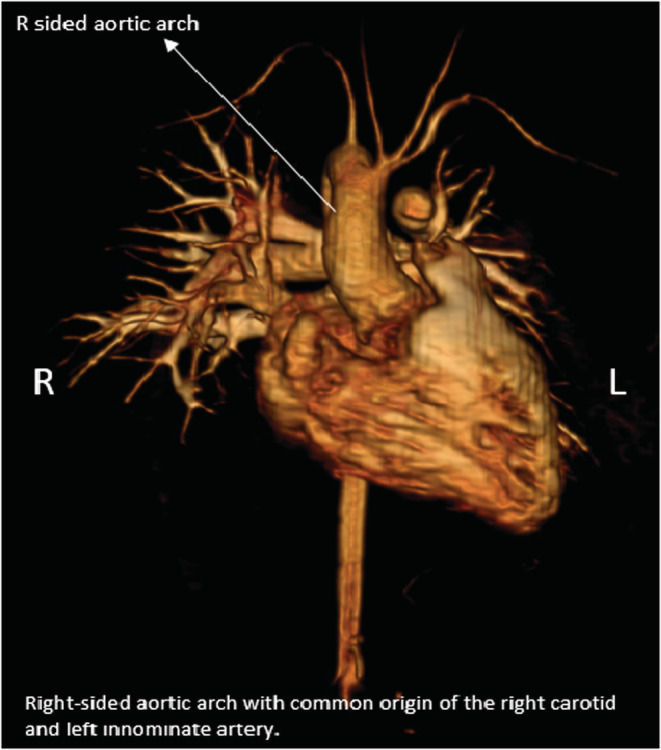
Figure shows the right aortic arch with a common origin of the right carotid and left innominate artery

On examination, vitals were stable with a pulse rate of 70 beats per min, blood pressure 112/76 mm Hg, the body temperature of 37.7 C, respiratory rate of 18 breaths per min, and oxygen saturation of 100%. Physical examination revealed a holo‐diastolic murmur at the left sternal border. Systemic examination including pulmonary, abdominal, neurological, and musculoskeletal was otherwise unremarkable. Blood counts and metabolic panel were normal. Chest X‐ray showed mild cardiomegaly and a right‐sided aortic arch. Electroencephalogram (EKG) showed the right bundle branch block. An echocardiogram revealed mild pulmonic valve stenosis and severe insufficiency, normal right ventricle size, and function. Chest magnetic resonance angiography showed a right‐sided aortic arch with a common origin of the right carotid and left innominate artery with normal‐sized aortic root and thoracic aorta as shown in Figure 1 below.

**FIGURE 2:**
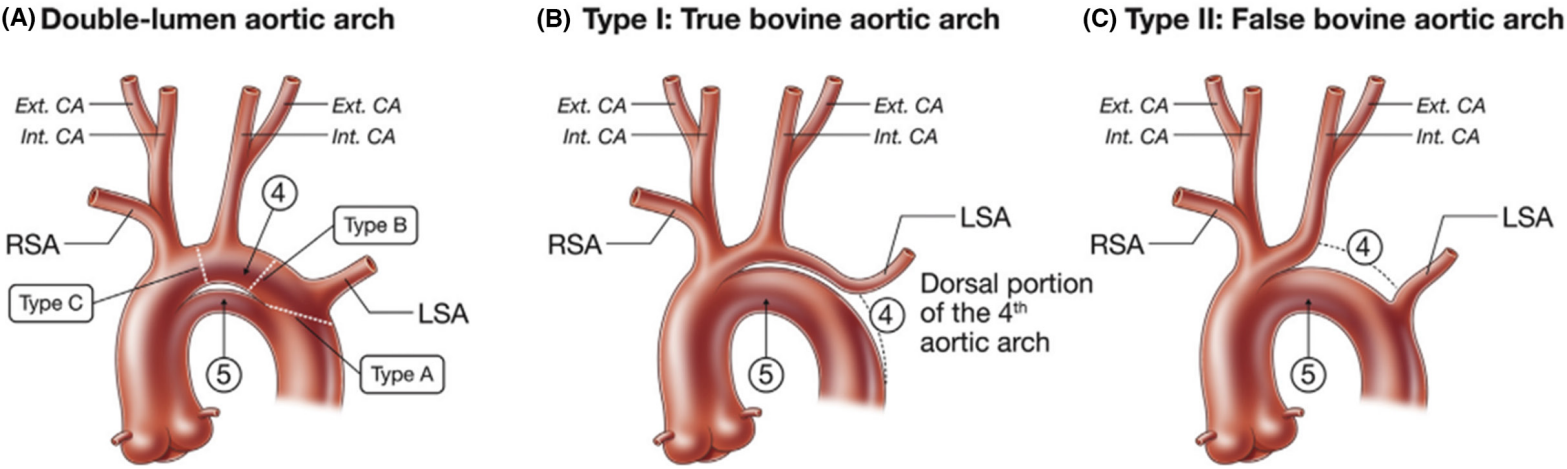
Types of right‐sided aortic arch.^12^

The treatment option of surgical vs percutaneous pulmonic valve replacement was discussed. Due to high surgical risk and normal right ventricle size, surgery was deferred at this point. She continues to remain under active surveillance with periodic follow‐up.

## 3 DISCUSSION

Aortic arch development is a complex process that begins during the third week of gestation and involves six pairs of arches. Failure of normal development, segmentation, or regression of these embryonic aortic arches leads to multiple aortic arch anomalies ranging from asymptomatic variations in arch vessel patterns to a variety of symptomatic pathological anomalies. The prevalence of aortic arch anomalies is between 0.5 and 3.0%.^5^ Aortic arch anomalies are commonly associated with congenital heart defects, chromosomal abnormalities, and esophageal or tracheal compression symptoms.^6^, ^7^, ^8^

RAA occurs when the aortic arch traverses over the right bronchus instead of the left and descends along the right side of the spine. The RAA is present in 0.01%–0.1% of the general population. Fioretti and Aglietti first reported this anomaly at autopsy in 1763.^9^ Embryologically, the left fourth aortic arch forms the adult aortic arch while the right generally regresses resulting in a normal left aortic arch descending along the left side of the spine; however, if the left arch regresses and the right persists, it leads to the development of RAA.^3^

In a study by Prabhu et al., 10 variations of the right aortic arch, which arises from the fourth right pharyngeal arch artery, were identified, with potential embryological causes.^10^ This classification excludes right arches originating from structures other than the right fourth pharyngeal arch vessel, such as the right‐sided cervical aortic arch, as well as right arches containing the circumflex aorta. The classification remains the same whether a right‐sided arterial duct is present or not. The explanation is based on Edwards' theory of the double aortic arch and Rathke's depiction of aortic arches.

RAA does not cause any physiological cardiovascular effects by itself; however, it is commonly associated with various congenital heart diseases including TOF, truncus arteries, tricuspid atresia, and transposition of great arteries.^11^ RAA anomalies are usually benign and remain asymptomatic unless they develop aneurysmal disease‐causing symptoms of airway compression, esophageal compression, heart failure, or abnormal imaging findings and can present at any age.

The clinician must have a high degree of suspicion to diagnose anomalies related to the aortic arch because they are relatively uncommon, and asymptomatic but associated with congenital heart disease and aneurysmal complications. Diagnostic evaluation includes Chest X‐ray, echocardiography, CT angiography (CTA), and MR angiography (MRA). Chest X‐ray determines the orientation of the aortic arch. Echocardiography helps identify any associated cardiac structural abnormalities. CTA/MRA can identify the definitive branching patterns.

The patient described had a right‐sided aortic arch with a common origin of right carotid and left innominate artery, which is a different variant, compared to the ones described before. The patient was asymptomatic from this particular anomaly itself, however, had associated TOF, POF, and pulmonary stenosis that were symptomatic. She had a surgical repair for TOF, POF, and stenting for pulmonary stenosis. The current symptoms were due to pulmonary insufficiency. Due to the high surgical risk, the patient had medical management with close periodic follow‐up.

To our knowledge, this is the first case reporting RAA with a common origin of right carotid and left innominate artery. The associated congenital abnormalities with this variant include TOF, PFO, and pulmonary stenosis. Management for the arch anomaly depends upon symptoms and can range from close monitoring to surgical repair for complications. It is important to recognize aortic arch anomalies as they can be associated with various congenital heart diseases, and chromosomal anomalies and can have implications for prognostication and management.

## 4 CONCLUSION

We report the first case in the literature of a right‐sided aortic arch with a common origin of right carotid and left innominate artery. The patients may primarily be asymptomatic from this aortic arch anomaly but may develop symptoms from the associated congenital heart disease or from a complication of aneurysm formation. Prior to this case report, three variants of RAA based on branching patterns are known.

## AUTHOR CONTRIBUTIONS

SS, SC, and ST wrote the original manuscript, reviewed, and edited the manuscript. OB, DR, JD, and BB reviewed and edited the original manuscript and were in charge of the case.

## CONFLICT OF INTEREST

None.

## ETHICAL APPROVAL

None.

## CONSENT

Written informed consent was obtained from the patient for publication of the case report.

## Data Availability

All the required information are in manuscript itself
